# Metatranscriptome analysis in human papillomavirus negative cervical cancers

**DOI:** 10.1038/s41598-022-19008-8

**Published:** 2022-09-05

**Authors:** Agustin Enrique Ure, Camilla Lagheden, Laila Sara Arroyo Mühr

**Affiliations:** Department of Laboratory Medicine, Karolinska Institutet, 141 86, Stockholm, Sweden

**Keywords:** Virology, Microbiology, Molecular biology, Cancer, Tumour virus infections

## Abstract

Human papillomavirus (HPV) negative cancers are associated with symptomatic detection, late-stage diagnosis, and worse prognosis. It is thus essential to investigate all possible infectious agents and biomarkers that could early identify these HPV negative cancers. We aimed to analyze and compare the metatranscriptome present in HPV positive and HPV negative cervical cancers. We analyzed the whole RNA sequencing files from 223 HPV negative cervical cancers (negativity established after confirming cervical cancer diagnosis, sample adequacy and subjecting specimens to PCR and unbiased RNA sequencing), 223 HPV positive tumors and 11 blank paraffin block pools (used as controls) using Kraken2 software. Overall, 84 bacterial genera were detected, with 6/84 genera showing a positive median number of reads/sample and being present in both cervical tumor groups (HPV positive and negative). Viral reads belonged to 63 different viral genera, with 6/63 genera showing a positive median annotated read/sample value. No significant difference among genera was detected except for the presence of alpha-papillomaviruses. Metatranscriptome of bacteria and viruses present in HPV positive and HPV negative cervical cancers show no significant difference, except for HPV. Further studies are needed to early identify this biologically distinct group of cervical cancers.

## Introduction

Human papillomaviruses are a group of double-stranded DNA viruses which comprise up to 223 different types, with new types being continuously identified^1–4^. About 12 HPV types are nowadays classified as oncogenic, high-risk HPV genotypes, and persistent infection of these oncogenic HPV types is known to be a necessary cause for cervical cancer.

Although effective screening methods (cytology and HPV testing) exist and several effective prophylactic vaccines are available in the market to prevent cervical cancer, the annual number of new cases of cervical cancer is estimated to increase from 570,000 to 700,000 between 2018 and 2030, with the annual number of deaths rising from 311,000 to 400,000^5^. Cervical cancer is preventable, but it is still one of the most common cancers and causes of cancer-related death in women, worldwide.

Due to advances in molecular testing, the more sensitive method of HPV-based screening is now replacing cytology as the primary cervical cancer screening tool. This method translates into testing cervical samples for HPV DNA rather than morphological abnormalities and constitute a more automated and objective screening strategy. Women with a negative HPV test, have very low risk of subsequent cancer and may safely be re-called in three-seven years. Nevertheless, a significant proportion of cervical cancers (around 7%) appears to be HPV negative^6–8^. This percentage of HPV negative cancers may translate into women not being categorized correctly for the screening program and tumors being detected only when they had become symptomatic (very late). Of particular note, authors have shown that cervical cancer cases that appear to have lost HPV DNA seem to constitute a specific subgroup of cervical cancers with a different biological behavior (worse prognosis)^9,10^.

Efforts to find out and explain HPV negativity in cervical cancers have led scientists to reanalyze smears and retest these HPV negative tumors by using different approaches. Main explanations for HPV negativity have been sample inadequacy, false diagnosis, presence of cancers associated with types that were not targeted in the studies, and false negative results due to low sensitivity of the HPV detection methods^6–8,11^.

Most HPV screening methods are focused on detecting high-risk HPV types and are based on PCR amplification of conserved regions in the L1 gene by consensus primers, followed by HPV genotyping identification by hybridization of amplicons to type-specific probes^12,13^. However, these methods are biased to detect mostly known HPVs that bind specifically to designed primers and probes. Unknown HPV types, known HPV types that are distantly related as well as targeted HPV types presenting variations within probe/primer regions may escape amplification or hybridization^4,14–17^. To overcome this bottleneck, it is nowadays possible to perform unbiased metagenomic sequencing (not based on PCR) and detect all HPVs present in a sample, without prior knowledge on which types might be present^14,18,19^. Furthermore, if cDNA is sequenced, the data will reveal if there is viral transcriptional activity, essential for both initiation and maintenance of the malignant phenotype.

Following the three recommendations mentioned above, cervical tumors classified as HPV negative after (a) assuring sample adequacy, (b) reanalysis of corresponding slides/smears to confirm tumor diagnosis and (c) not revealing presence of HPV after performing an unbiased sequencing analysis, may be called truly HPV negative. Understanding the biology of truly HPV negative cervical cancers is urgently needed in the era of cervical cancer elimination.

Cervical cancer is known to have an infection as cause, being HPV the primary predisposing factor. Truly HPV negative cervical cancers could have initially been caused by HPV but lost the virus as the tumor progressed. There is a very high proportion of cervical intraepithelial neoplasias (CIN) 3 that contains HPV and there are cases of HPV negative cancers that have been HPV positive in the earlier screening appointments^20^. On the other hand, it is possible that another infectious agent may play a role in this specific subgroup of HPV negative cancers. Taking into consideration both possibilities, a search for an infectious cause/biomarker should focus on unbiased RNA sequencing of truly HPV negative cancers. We aimed to analyze and compare the metatranscriptome of HPV positive cervical cancers with the metatranscriptome of HPV negative cancers and assess whether there are differences that could act as biomarkers to early identify these HPV negative cancers.

## Materials and methods

In a previous study, we systematically genotyped all cervical cancer cases occurring in Sweden from 2002–2011 (n = 2850), and for those that were HPV negative (n = 527, 18.5% of total cervical tumors), sample adequacy and correct diagnosis were analyzed and reanalyzed, respectively. After excluding samples that were not adequate (beta-globin negativity) and those whose diagnosis was not confirmed, a different PCR approach (targeting *E6/E7* genes instead of *L1*) was performed. Repeatedly negative samples decreased to 394/2850 (13.8% of total cervical tumors). We then subjected all HPV negative carcinomas (and a subset of 59 HPV PCR positive cervical cancers used as positive controls) to an unbiased RNA sequencing using Novaseq 6000 (Illumina Platform), generating high quality sequencing data with a median of 30 million reads per sample. RNA positivity was detected in 169/392, decreasing the percentage of cervical cancers negative for HPV from 18.5% to 7.8% (223/2850)^15^.

For this study, we selected all the 223 HPV negative specimens and compared the metatranscriptomes present with the metatranscriptomes detected in 223 HPV positive cervical cancers. These 223 HPV positive cervical specimens corresponded to 169 specimens that were originally HPV PCR negative but turned out to be HPV positive when subjected to unbiased sequencing as well as 54/59 HPV PCR positive samples used previously as positive controls (5/59 HPV PCR positive samples were HPV negative when subjected to sequencing). A total of 11 blank paraffin blocks pools were used as negative controls. While sectioning each cervical tumor formalin-fixed paraffin-embedded (FFPE) block, blank paraffin blocks had been sectioned in between as control for contamination. These paraffin blocks were extracted in the same manner as the cervical tumors, pooled (each pool containing about 45 blank blocks) and sequenced together with the corresponding tumor blocks.

High-quality non-human reads were classified using Kraken2 v. 2.1.1^21^, which was run against a reference database containing all RefSeq bacterial and viral genomes (built in December 2020) with a 0.1 confidence threshold. A cut-off of 10 classified unique reads was used to discriminate positive genera for bacteria and viruses, and results reported all genera which comprised more than 1% of total bacterial or viral reads, respectively.

As HPV was the key difference between the cervical tumors (and the only significant difference detected between the 2 groups), we also queried all high-quality non-human reads to a reference database containing all human papillomavirus nucleotide sequences deposited in GenBank until July 8th 2022 (parameters: Taxid 151,340, length 5000–12,000, excluding non-partial genomes) using Kraken2 v. 2.1.1^21^, with a 0.1 confidence threshold. A cut-off of 10 classified unique reads was used to discriminate positive species. Reads that corresponded to HPV genomes were subjected to visual inspection using Integrative Genomics Viewer to confirm mapping.

### Statistical analysis

Differences in bacterial/viral presence across the two groups (HPV positive and HPV negative cancers) were evaluated by comparing median unique reads with nonparametric Wilcoxon rank-sum test, and relative proportions of bacteria/viral communities among the HPV positive and HPV negative cervical cancers were analyzed using a two proportion Z-test and its associated p-value. Bonferroni correction was applied considering a 0.05 error rate (alpha level) and statistical significance was then obtained if p < 0.0002.

Ethical approval was granted by the Regional Ethical Review Board of Stockholm, Sweden (EPN-Dnr: 2012/1028/32). The Regional Ethical Review Board determined that, due to the population-based nature of the study, informed consent from study participants was not required (EPN-Dnr: 2011/1026–31/4). All methods were performed in accordance with the relevant guidelines and regulations.

## Results

In a previous study, all cervical cancers diagnosed from 2002 to 2011 in the whole Sweden were collected and genotyped. HPV negativity was determined after sample adequacy confirmation, diagnose reanalysis and confirmation of cervical cancer, and not detecting HPV after both PCR and unbiased RNA sequencing^15^. A total of 223 HPV negative cervical cancers were reported. The present study aimed to compare the metatranscriptome present in these 223 HPV negative cervical cancer specimens with the corresponding metatranscriptome present in 223 HPV positive cervical tumors.

The median age at cancer diagnosis of the HPV negative cervical cases (n = 223) was 68 years (range 30–93 years) and the median age at cancer diagnosis of the HPV positive cervical cases (n = 223) was 56 years (range 24–95 years).

### Bacteriome

The metatranscriptome analysis from RNA sequencing had a total number of 63.80 M annotated bacterial reads for the HPV positive specimens and 62.81 M annotated bacterial reads for the HPV negative specimens.

A total of 84 different bacterial genera showing at least 10 reads and 1% of total bacterial annotated reads were detected within the 223 HPV positive and 223 HPV negative cervical tumors. Only 6/84 genera showed a positive median number of reads/sample for both specimen groups (HPV positive and HPV negative cancers) (Table 1).

**Table 1:** Bacterial genera and annotated reads detected in cervical cancer specimens.

Bacteria genera	HPV positive samples (Total 223 samples)				HPV negative (Total 223 samples)				p-value (difference in	p-value (difference in	HPV blanks (Total 11 pools)			
	Median	Min	Max	N	Median	Min	Max	N	read median level)	proportion positive)	Median	Min	Max	N
Acetoanaerobium	0	0	2797	1	0	0	0	0	0.9350	0.3170	0	0	0	0
Acidovorax	0	0	15,747	5	0	0	34,892	6	0.9340	0.7598	32,774	0	352,979	7
Acinetobacter	0	0	63,612	35	0	0	44,938	32	0.5915	0.6910	30,671	0	460,997	10
Actinotignum	0	0	0	0	0	0	15,765	4	0.7431	0.0445	0	0	0	0
Alistipes	0	0	3315	2	0	0	0	0	0.8699	0.1563	0	0	0	0
Allopseudarcicella	0	0	9664	2	0	0	57,011	4	0.8693	0.4110	0	0	398,225	5
Anaerococcus	0	0	17,749	3	0	0	5474	3	0.9991	1.0000	0	0	0	0
**Bacillus**	2898	0	14,526	196	3260	0	19,284	196	0.1421	1.0000	0	0	39,783	3
Bacteroides	0	0	1,506,030	12	0	0	203,682	18	0.6290	0.2566	0	0	0	0
Belliella	0	0	2009	1	0	0	0	0	0.9347	0.3170	0	0	0	0
Bifidobacterium	0	0	8321	1	0	0	0	0	0.9347	0.3170	0	0	0	0
Blautia	0	0	0	0	0	0	22,243	1	0.9347	0.3170	0	0	0	0
Bradyrhizobium	0	0	10,157	3	0	0	7445	1	0.8696	0.3150	0	0	0	0
**Burkholderia**	5633	0	44,906	215	5643	0	36,944	218	0.8742	0.3985	0	0	113,468	5
Campylobacter	0	0	103,670	13	0	0	52,901	11	0.8701	0.6747	0	0	0	0
Candidatus Planktophila	0	0	26,630	4	0	0	117,273	8	0.7415	0.2420	0	0	868,218	5
Cloacibacterium	0	0	0	0	0	0	4061	1	0.9347	0.3170	0	0	207,611	2
Clostridium	0	0	6593	3	0	0	34,166	7	0.7390	0.2007	2679	0	302,707	6
Collimonas	0	0	5656	1	0	0	2297	1	0.9997	1.0000	0	0	0	0
Corynebacterium	0	0	12,996	1	0	0	15,676	1	0.9997	1.0000	0	0	1,140,316	2
Cruoricaptor	0	0	0	0	0	0	22,557	1	0.9347	0.3170	0	0	0	0
Cutibacterium	0	0	23,277	4	0	0	4880	6	0.8751	0.5224	0	0	99,667	1
Desulfobulbus	0	0	2374	1	0	0	0	0	0.9347	0.3170	0	0	0	0
Desulfovibrio	0	0	3566	1	0	0	0	0	0.9347	0.3170	0	0	0	0
Dietzia	0	0	12,914	6	0	0	3495	1	0.6815	0.0574	0	0	0	0
Dyadobacter	0	0	1288	1	0	0	0	0	0.9347	0.3170	0	0	0	0
Enterocloster	0	0	475,262	2	0	0	14,351	1	0.9347	0.5618	0	0	0	0
Enterococcus	0	0	1228	1	0	0	5404	2	0.9341	0.5618	0	0	0	0
Escherichia	0	0	19,460	55	0	0	36,466	49	0.8318	0.5029	0	0	3335	1
Ethanoligenens	0	0	15,494	1	0	0	0	0	0.9347	0.3170	0	0	39,819	1
Ezakiella	0	0	10,140	2	0	0	9552	4	0.8704	0.4110	0	0	0	0
Fannyhessea	0	0	30,036	1	0	0	6555	1	0.9997	1.0000	0	0	0	0
Fastidiosipila	0	0	2021	1	0	0	30,069	2	0.9341	0.5618	0	0	0	0
Filifactor	0	0	11,696	1	0	0	0	0	0.9347	0.3170	0	0	0	0
Flavobacterium	0	0	58,987	14	0	0	128,908	29	0.2213	0.0160	164,299	0	1,767,013	10
Fusobacterium	0	0	273,120	14	0	0	610,444	12	0.8814	0.6860	0	0	0	0
Gardnerella	0	0	325,008	12	0	0	72,179	2	0.4122	0.0066	0	0	0	0
Halomonas	0	0	11,206	23	0	0	27,156	32	0.4914	0.1949	38,767	0	426,492	10
Hungatella	0	0	0	0	0	0	37,581	1	0.9347	0.3170	0	0	0	0
Hydrogenophaga	0	0	21,079	79	0	0	21,465	83	0.7301	0.6937	0	0	95,973	1
Janthinobacterium	0	0	6290	2	0	0	21,719	3	0.9341	0.6532	0	0	136,896	5
Jonquetella	0	0	0	0	0	0	48,322	1	0.9347	0.3170	0	0	0	0
Kinneretia	0	0	0	0	0	0	1803	1	0.9347	0.3170	0	0	0	0
**Klebsiella**	66,563	0	526,334	222	69,618	0	430,646	222	0.6932	1.0000	0	0	120,216	1
Lacrimispora	0	0	0	0	0	0	20,296	1	0.9347	0.3170	0	0	0	0
Lactobacillus	0	0	643,227	7	0	0	6916	3	0.7395	0.2007	0	0	0	0
Limnohabitans	0	0	14,587	6	0	0	57,491	7	0.9370	0.7786	48,533	0	549,022	10
Mageeibacillus	0	0	109,370	4	0	0	0	0	0.7431	0.0445	0	0	0	0
Massilia	0	0	9937	2	0	0	3169	1	0.9341	0.5618	0	0	2142	1
Melittangium	0	0	7951	1	0	0	0	0	0.9347	0.3170	0	0	0	0
Methylobacterium	0	0	7041	1	0	0	2952	1	0.9997	0.3170	0	0	0	0
Microbacterium	0	0	0	0	0	0	2537	2	0.8699	0.1563	0	0	569,938	2
Mobiluncus	0	0	16,201	1	0	0	6498	2	0.9353	0.5618	0	0	0	0
Neisseria	0	0	0	0	0	0	435,366	2	0.8699	0.1563	0	0	0	0
Nesterenkonia	0	0	7946	5	0	0	3846	2	0.8059	0.2532	0	0	0	0
Nocardia	0	0	0	0	0	0	2921	2	0.8699	0.1563	0	0	0	0
Nonlabens	0	0	1655	1	0	0	0	0	0.9347	0.3170	0	0	0	0
Parabacteroides	0	0	1280	1	0	0	0	0	0.9347	0.3170	0	0	0	0
Paraburkholderia	0	0	20,221	62	0	0	23,135	60	0.8433	0.8337	0	0	0	0
**Paracoccus**	4817	0	36,916	211	4955	0	30,106	212	0.6810	0.8306	0	0	8812	1
Paraglaciecola	0	0	1144	1	0	0	1777	1	0.9997	1.0000	0	0	0	0
Parvimonas	0	0	0	0	0	0	3117	1	0.9347	0.3170	0	0	0	0
**Pasteurella**	31,263	0	239,268	222	31,026	0	240,531	222	0.7084	1.0000	0	0	53,991	1
Peptoniphilus	0	0	16,722	8	0	0	7424	8	0.9971	1.0000	0	0	0	0
Phocaeicola	0	0	57,525	2	0	0	7305	1	0.9341	0.5617	0	0	0	0
Polaromonas	0	0	3543	1	0	0	10,144	3	0.8696	0.3173	0	0	110,408	1
Polynucleobacter	0	0	10,111	7	0	0	42,334	8	0.9370	0.7930	34,868	0	337,142	10
Porphyromonas	0	0	594,881	25	0	0	483,504	28	0.8047	0.6607	0	0	0	0
Prevotella	0	0	138,942	19	0	0	120,686	13	0.6327	0.2711	0	0	0	0
Pseudomonas	0	0	71,558	106	0	0	59,681	93	0.2141	0.2156	46,800	12,837	415,299	11
Ralstonia	0	0	23,359	81	0	0	9351	59	0.1233	0.0248	0	0	0	0
Rhodoluna	0	0	40,386	11	0	0	164,642	16	0.6815	0.3209	114,403	0	1,285,848	10
Roseburia	0	0	2071	1	0	0	0	0	0.9347	0.3170	0	0	0	0
Selenomonas	0	0	1619	1	0	0	0	0	0.9347	0.3170	0	0	0	0
Sneathia	0	0	24,953	2	0	0	0	0	0.8699	0.1563	0	0	0	0
Sphingomonas	0	0	10,020	4	0	0	2893	3	0.9324	0.7029	0	0	135,002	3
**Staphylococcus**	40,020	0	315,114	222	39,935	0	254,654	222	0.7934	1.0000	0	0	71,438	1
Streptococcus	0	0	26,160	6	0	0	6776	3	0.8059	0.3121	0	0	0	0
Streptomyces	0	0	8088	25	0	0	14,558	23	0.8413	0.7599	0	0	0	0
Tannerella	0	0	2673	2	0	0	5034	1	0.9353	0.5617	0	0	0	0
Tenuifilum	0	0	1686	1	0	0	0	0	0.9347	0.3170	0	0	0	0
Thermoanaerobacterium	0	0	5416	2	0	0	3423	1	0.9327	0.5617	0	0	0	0
Treponema	0	0	37,987	4	0	0	6113	2	0.8693	0.4110	0	0	0	0
Xanthomonas	0	0	2399	4	0	0	5605	3	0.9365	0.7029	0	0	0	0

Bacterial genera highlighted in bold correspond to genera presenting a positive median number of annotated reads/sample. Genera are reported when comprising at least 10 reads and being at least 1% of total bacterial reads/sample.

The same 6 genera were found in both types of cervical tumors (HPV positive and HPV negative cancers), and the corresponding proportions were similar. From higher to lower number of median annotated reads/sample: *Klebsiella* (66,563 and 69,618 median reads/sample), *Staphylococcus* (40,020 and 39,935 reads), *Pasteurella* (31,263 and 31,026 reads), *Burkholderia* (5633 and 5643 reads), *Paracoccus* (4817 and 4955 median reads) and Bacillus (2898 and 3260 reads) were the genera detected in HPV positive and HPV negative specimens, respectively. All 6 genera were present in at least 87.89% of specimens. No statistical difference (p < 0.001) in median read level nor in proportion was detected when comparing any bacteria genera within HPV positive and HPV negative specimens (Table 1).

Analysis of controls (blank paraffin specimens) showed a total of 31 different genera with 28/31 genera being also detected in cervical cancer specimens. All 6 genera that showed positive median read values in cervical tumors, were also present in at least one of the blank controls, with *Klebsiella*, *Staphylococcus*, *Pasteurella and Paracoccus being identified in 1/11 blank controls and Burkholderia* and Bacillus in 5 and 3 blank controls, respectively.

### Virome

The metatranscriptome analysis from RNA sequencing had a total number of 613,606 annotated viral reads for the HPV positive specimens and 575,734 annotated viral reads for the HPV negative specimens.

Overall, 63 different viral genera were detected when analyzing the RNA metatranscriptome within HPV positive and HPV negative tumors, with 6/28 genera showing a positive median annotated read/sample value (Table 2).

**Table 2:** Viral genera and annotated reads detected in cervical cancer specimens.

Viral genera	HPV positive samples (Total 223 samples)				HPV negative (Total 223 samples)				p-value (difference in	p-value (difference in	HPV blanks (Total 11 pools)			
	Median	Min	Max	N	Median	Min	Max	N	read median level)	proportion positive)	Median	Min	Max	N
Acionnavirus	0	0	10	1	0	0	0	0	0.9347	0.3170	0	0	0	0
**Alphabaculovirus**	14	0	121	122	0	0	145	107	0.2610	0.1553	0	0	170	2
**Alphapapillomavirus**	34	0	19,879	142	0	0	40	3	0.0000	0.0000	0	0	54	2
Alpharetrovirus	0	0	52	1	0	0	0	0	0.9347	0.3170	0	0	0	0
Alphatectivirus	0	0	436	1	0	0	0	0	0.9347	0.3170	0	0	0	0
Alphatorquevirus	0	0	0	0	0	0	77	1	0.9347	0.3170	0	0	0	0
Andhravirus	0	0	58	10	0	0	245	10	0.9977	1.0000	0	0	0	0
Aresaunavirus	0	0	13	1	0	0	0	0	0.9347	0.3170	0	0	0	0
**Betabaculovirus**	16	0	241	133	21	0	223	140	0.5117	0.4964	365	15	3364	11
Betatorquevirus	0	0	0	0	0	0	17	1	0.9347	0.3170	0	0	0	0
Bjornvirus	0	0	10	1	0	0	0	0	0.9347	0.3170	0	0	154	2
Bronvirus	0	0	14	1	0	0	20	1	0.9997	1.0000	0	0	0	0
Caeruleovirus	0	0	108	82	0	0	166	85	0.7199	0.7690	0	0	232	2
Casadabanvirus	0	0	0	0	0	0	41	1	0.9347	0.3170	0	0	0	0
Ceetrepovirus	0	0	12	1	0	0	16	1	0.9997	1.0000	0	0	1414	3
Centapoxvirus	0	0	15	2	0	0	22	2	1.0000	1.0000	0	0	0	0
Cheoctovirus	0	0	0	0	0	0	10	1	0.9347	0.3170	0	0	33	2
Chlorovirus	0	0	14	2	0	0	25	3	0.9344	0.6532	61	0	537	9
Coralvirus	0	0	0	0	0	0	12	1	0.9347	0.3170	0	0	111	1
Cyprinivirus	0	0	17	7	0	0	22	4	0.8059	0.3598	0	0	0	0
Delepquintavirus	0	0	0	0	0	0	11	1	0.9347	0.3170	0	0	436	3
Dyokappapapillomavirus	0	0	68	1	0	0	0	0	0.9347	0.3170	0	0	0	0
Dyopipapillomavirus	0	0	51	2	0	0	0	0	0.8699	0.1563	0	0	0	0
Fipvunavirus	0	0	32	1	0	0	10	1	0.9997	1.0000	0	0	144	1
Gammacarmovirus	0	0	0	0	0	0	13	1	0.9347	0.3170	0	0	0	0
Gammapapillomavirus	0	0	54	1	0	0	0	0	0.9347	0.3170	0	0	0	0
Gammaretrovirus	0	0	330	43	0	0	271	34	0.4697	0.2595	0	0	0	0
Gemycircularvirus	0	0	178	8	0	0	101	5	0.8116	0.3985	70	0	832	9
Gemygorvirus	0	0	0	0	0	0	24	1	0.9347	0.3170	0	0	166	5
Gemykibivirus	0	0	12	1	0	0	38	4	0.8050	0.1770	0	0	331	4
**Gorganvirus**	694	0	14,436	222	833	0	11,430	222	0.2902	1.0000	0	0	705	3
Iodovirus	0	0	0	0	0	0	13	1	0.9347	0.3170	0	0	142	1
Kalppathivirus	0	0	59	1	0	0	13	3	0.8707	0.3150	0	0	1545	5
Kochikohdavirus	0	0	14	1	0	0	0	0	0.9347	0.3170	0	0	0	0
Korravirus	0	0	10	1	0	0	0	0	0.9347	0.3170	0	0	0	0
Locarnavirus	0	0	73	5	0	0	38	4	0.9306	0.7366	29	0	861	6
Lymphocryptovirus	0	0	125	6	0	0	53	4	0.8699	0.5224	0	0	0	0
Majavirus	0	0	0	0	0	0	14	1	0.9347	0.3170	0	0	0	0
Mardivirus	0	0	83	54	0	0	112	57	0.7993	0.7424	0	0	0	0
Mimoreovirus	0	0	0	0	0	0	10	1	0.9347	0.3170	0	0	0	0
Mupapillomavirus	0	0	40	6	0	0	0	0	0.6230	0.0137	0	0	0	0
**Orthobunyavirus**	566	0	6583	210	608	0	9192	213	0.0981	0.5204	257	0	3116	10
Otagovirus	0	0	0	0	0	0	12	1	0.9347	0.3170	0	0	107	1
Pahexavirus	0	0	233	7	0	0	62	8	0.9382	0.7930	0	0	600	3
**Pandoravirus**	13	0	163	119	0	0	188	109	0.4720	0.3435	0	0	132	3
Percavirus	0	0	19	6	0	0	14	2	0.7431	0.1535	0	0	0	0
Phietavirus	0	0	0	0	0	0	18	1	0.9347	0.3170	0	0	0	0
Prasinovirus	0	0	27	1	0	0	21	1	0.9997	1.0000	0	0	66	1
Proboscivirus	0	0	16	1	0	0	19	3	0.8701	0.3150	0	0	0	0
Prymnesiovirus	0	0	12	2	0	0	0	0	0.8699	0.1563	0	0	0	0
Ranavirus	0	0	10	1	0	0	0	0	0.9347	0.3170	0	0	0	0
Rimavirus	0	0	0	0	0	0	18	1	0.9347	0.3170	0	0	0	0
Roseolovirus	0	0	928	1	0	0	0	0	0.9347	0.3170	0	0	0	0
Salmondvirus	0	0	0	0	0	0	12	1	0.9347	0.3170	0	0	0	0
Seadornavirus	0	0	0	0	0	0	15	1	0.9347	0.3170	0	0	164	2
Simplexvirus	0	0	19	1	0	0	30	1	0.9997	1.0000	0	0	0	0
Skunavirus	0	0	105	1	0	0	13	1	0.9997	1.0000	0	0	24	1
Sobemovirus	0	0	19	1	0	0	12	1	0.9997	1.0000	0	0	332	4
Terapinvirus	0	0	0	0	0	0	16	1	0.9347	0.3170	0	0	65	1
Titanvirus	0	0	0	0	0	0	10	1	0.9347	0.3170	0	0	325	2
Tombusvirus	0	0	0	0	0	0	18	1	0.9347	0.3170	0	0	122	3
Whispovirus	0	0	20	2	0	0	13	2	0.9988	1.0000	0	0	69	1
Yuavirus	0	0	0	0	0	0	10	1	0.9347	0.3170	0	0	0	0

Viral genera highlighted in bold correspond to genera presenting a positive median number of annotated reads/sample. Genera are reported when comprising at least 10 reads and being at least 1% of total viral reads/sample.

Three out of these 6 genera*: Gorganvirus, Orthobunyavirus and Betabaculovirus,* showed a positive median read value in both HPV negative and HPV positive cervical tumors while *Alphabaculovirus, Alphapapillomavirus and Pandoravirus* showed a positive median read value only in HPV positive tumors. Statistical significance (p < 0.001) was only detected for the *Alphapapillomavirus* genus (both when analysing the difference in median unique reads as well as the relative proportions). *Alphapapillomavirus* genus was detected in 142/223 HPV positive specimens and in 3/223 HPV negative specimens according to established cutoffs and reporting parameters (10 reads and at least 1% of total viral reads).

Further analysis of HPV presence was performed by subjecting high-quality non-human reads to a more “complete” database containing thousands of human papillomavirus sequences deposited in GenBank using Kraken. The analysis revealed HPV positivity (considered when samples showed more than 10 reads/species) in all 223 HPV positive cervical tumors, 7/10 controls blank pools and 30/223 HPV negative cervical tumors. Visual inspection of reads using Integrative Genomics Viewer showed that the blank controls and HPV negative cervical tumors that turned out to be HPV positive with this analysis did in fact show more than 10 reads, but reads were very short (< 50 bp) and viral coverage was below 10%. All 223 HPV positive cervical tumors showed more than 10 reads and HPV coverage was above 10% for all specimens.

Analysis of controls (blank paraffin specimen pools) revealed presence of 74 different viral genera, with 30/74 genera being also present in HPV negative and/or HPV positive cervical cancer samples. Most of the genera (25/30) were present in less than half of the blank controls, while *Locarnavirus, Chlorovirus, Gemycircularvirus, Orthobunyavirus* and *Betabaculovirus*, were present in 6/11, 9/11, 9/11, 10/11 and 11/11 blank pools, respectively.

## Discussion

Cervical HPV negative cancers exist and have unique properties such as late stage diagnosed cancers with poor prognosis. In this paper, we compared the metatrascriptome from 223 HPV negative cervical cancers with the metatranscriptome corresponding to 223 HPV positive cancers to inspect if there was any difference between them.

Strengths of this study include the use of HPV negative cancers whose diagnosis, sample adequacy and HPV result had been confirmed and analyzed with different methods in order to dismiss false negativity. FFPE specimens were reanalyzed by an expert pathologist and confirmed to indeed contain invasive cervical cancer tissue. Beta-globin was present in all specimens included in the study and HPV detection was performed by using PCR targeting *L1*, *E6/E7* as well as unbiased RNA sequencing. Furthermore, blank paraffin controls were used to assess possible contamination as well as presence of environmental communities, and Bonferroni correction was applied to prevent data from incorrectly appearing to be statistically significant (a common event when performing multiple comparisons).

Bacterial and viral communities have been already analyzed and compared between health and disease, specially thanks to the effort of the Human Microbiome Project (HMP), the first large study to address the diversity of microorganisms present in the different organs of the human body^22^. Literature agrees on reporting that “healthy” women show a vaginal microbiome with low diversity, dominated by *Lactobacillus*, and that one of the most prominent features of “disease” is an increase of pH (due to a decrease of lactate concentration), reduction of lactobacilli and a great diversity of bacterial vaginosis-related bacteria, which are primarily anaerobic bacteria^23^. Studies on HPV positive CIN and cervical cancers show higher diversity in vaginal microbiota, with depletion of *Lactobacillus crispatus* and increase abundance of anaerobic bacteria^24,25^. While our results from cervical cancer cases agree with the literature (higher diversity of bacteria and low abundance of Lactobacillus, only present in 10/446), microbiome differences between HPV negative and HPV positive cervical cancers have not been studied yet. No significant difference among present genera was detected except for the presence of alpha-papillomaviruses, which is in line with the fact that HPV is the only reported infectious agent so far associated with cervical cancer, supporting the hypothesis that loss of HPV may occur as the tumor progresses and may be the reason for the occurrence of such cancer which is almost 7% of all cervical cancers diagnosed. A weakness from the present study may comprise not having previous screening specimens from the corresponding women to see if HPV presence was detectable prior cancer diagnosis.

Human papillomaviruses were initially reported in 143/223 HPV positive cervical cancers (142 samples showing predominantly an alphapapillomavirus genus, and 1 specimen showing a *gammapapillomavirus*) and 3/223 HPV negative cervical cancers when querying sequencing reads to the RefSeq database. Genera were reported when they presented more than 10 reads and comprised at least 1% of total bacterial/viral reads/sample. A further investigation of HPV presence using a broader HPV database identified HPV in all HPV positive tumors, as well as in 7/10 controls blank pools and 30/223 HPV negative cervical tumors. Visual inspection of these reads revealed questionable calling for both the blank pools as well as the 30/223 HPV negative cervical tumors, where the viral coverage was below 10%.

There is no consensus about which cut-offs to use for HPV “calling” when performing sequencing analysis and there is an urgent need to establish those^26^. To reduce false-negative/positive classification, it is imperative to use complete and updated databases and take into consideration both the number of reads/k-mers (e.g. at least 10 reads) as well as the genome coverage to achieve accurate identification (e.g. at least 10%). Accepting number of reads/k-mers as only parameter is not enough, as artefacts/background/noise may produce false positivity^26,27^.

In conclusion, the present study reports the metatransciptome analysis of both HPV negative and HPV positive cervical tumors and does not detect any statistical difference in bacterial/viral communities´ expression when comparing HPV positive and HPV negative cervical cancers (except for *Alphapapillomavirus*). Further studies are needed to possibly find differences and/or biomarkers to early identify this biologically distinct subgroup of HPV negative cervical cancers. As the study is clear regarding that the metatranscriptome does not differ much between HPV negative and HPV positive cancers, we suggest that it may be more rewarding to look for differences in the human genome and transcriptome between the HPV positive and HPV negative cervical cancers.

## Data Availability

All the aligned, non-human sequences are available at the Sequence Read Archive (SRA) within the bio-project ID PRJNA563802 since previous publication.15 (https://www.ncbi.nlm.nih.gov/bioproject/563802).
